# Clinical utility of spectral analysis of intraocular pressure pulse wave

**DOI:** 10.1186/1471-2415-14-30

**Published:** 2014-03-12

**Authors:** Magdalena Asejczyk-Widlicka, Patrycja Krzyżanowska-Berkowska, Małgorzata Kowalska, D Robert Iskander

**Affiliations:** 1 Faculty of Materials, Strength and Welding, Wroclaw University of Technology, Smoluchowskiego 25, 50-372 Wroclaw, Poland; 2 Department of Ophthalmology, Wroclaw Medical University, Borowska 213, 50-556 Wroclaw, Poland; 3 Institute of Physics, Wroclaw University of Technology, Wybrzeze Wyspianskiego 27, 50-370 Wroclaw, Poland; 4 Institute of Biomedical Engineering and Instrumentation, Wroclaw University of Technology, Wybrzeze Wyspianskiego 27, 50-370 Wroclaw, Poland

**Keywords:** Glaucoma, Intraocular pressure, Dynamic contour tonometry, Ocular pulse wave

## Abstract

**Background:**

To evaluate the clinical utility of spectral analysis of intraocular pressure pulse wave in healthy eyes of a control group (CG), patients having glaucomatous optic disc appearance or ocular hypertension, and patients with primary open angle glaucoma or primary angle closure glaucoma.

**Methods:**

This is a prospective study that enrolled 296 patients from a single glaucoma clinic. Age matched CG consisted of 62 individuals. Subjects underwent comprehensive clinical diagnostic procedures including intraocular pressure (IOP) measurement with dynamic contour tonometry (DCT) and Goldmann applanation tonometry (GAT). DCT time series were analyzed with custom written software that included signal preprocessing, filtering and spectral analysis. An amplitude and energy content analysis, which takes into account non-stationarity of signals but also provides methodology that is independent of IOP and ocular pulse amplitude (OPA) levels, was applied. Spectral content up to the 6th harmonic of the pressure pulse wave was considered. Statistical analyses included descriptive statistics, normality test, and a multicomparison of medians for independent groups using Kruskal-Wallis test.

**Results:**

GAT IOP showed statistical significance (Kruskal-Willis test *p* < 0.05) for three out of 10 considered multiple comparisons, DCT IOP and OPA showed statistically significant results in five and seven cases, respectively. Changes in heart rate and central corneal thickness between the groups were statistically significant in two cases. None of the above parameters showed statistically significant differences between CG and the suspects with glaucomatous optic disc appearance (GODA). On the other hand, spectral analysis showed statistically significant differences for that case.

**Conclusions:**

Spectral analysis of the DCT signals was the only method showing statistically significant differences between healthy eyes and those of GODA suspects.

## Background

Cataract, age related macular degeneration and glaucoma are the leading causes of blindness in the developed countries. There are an estimated 60 million people with glaucomatous optic neuropathy and over 8 million blind people as the result of glaucoma [1]. In its early stages, glaucoma is asymptomatic and often difficult to diagnose. Measurement of the intraocular pressure (IOP) is regularly performed during early examination as well as follow-up of glaucoma patients. Although increased IOP is one of the risk factors in glaucoma, it is the vascular theory underlining blood supply deregulation that might be involved in the pathogenesis of glaucomatous optic neuropathy [2] and the corresponding structural and function loss that define it [3]. Nevertheless, currently the IOP seems to be the only modifiable factor in treating glaucoma [4,5].

Physiological fluctuations in IOP occur with the heart rate (HR). The difference between diastolic and systolic IOP is being referred to as the ocular pulse amplitude (OPA) [6-9]. It was reported that glaucoma patients display reduced OPA and reduced pulsatile ocular blood flow in comparison to control group of healthy subjects [10,11]. Those correlative results showed some potential of OPA as a diagnostic parameter, which currently is not routinely taken into account in diagnosis. Since IOP is utilized in glaucoma diagnosis and management, ideally, it should be obtained, as is the OPA, in a dynamic fashion from the recording of the intraocular pressure pulse wave.

Continuous pressure pulse wave can be directly measured with pneumatonometer or the dynamic contour tonometer (DCT, Pascal, Ziemer Ophthalmic System AG, Switzerland). In DCT, the pressure is measured directly on the external surface of the cornea, which according to the condition of matched contours [12,13] is theoretically equal to that inside the eye. DCT output contains a time series of several periods of the pressure pulse wave from which average diastolic IOP and maximum OPA values can be obtained. It was demonstrated that IOP parameters obtained from DCT are independent of geometrical and biomechanical properties of the cornea [12-14] but there are reports suggesting the contrary [15].

Evans and colleagues [16] were the first to report that spectral information of the intraocular pressure pulse wave, measured in their case with a pneumatonometer, can be utilized to distinguish glaucoma patients from normal subjects. In a small study of 10 normal subjects and 10 untreated glaucoma patients, they showed that the 2nd, 3rd and 4th harmonic components of the IOP pulse wave were significantly different between the groups. Recently, Božić and colleagues [17] have performed a similar study including a control group of 20 subjects and two groups of glaucoma patients including 20 with primary open angle glaucoma and 20 with normal tension glaucoma. There again, the spectral content of the intraocular pressure pulse wave, measured with DCT, showed differences between the groups.

The objective of this study was to ascertain, on a larger pool of subjects and patients than those used in studies of Evans and colleagues [16] and Božić and colleagues [17], whether spectral analysis of the intraocular pressure pulse wave has any added value to the traditional clinical techniques based on GAT and DCT.

## Methods

This prospective study included 296 participants (104 males and 192 females) enrolled from the regular patients of the Glaucoma Clinic at the Department of Ophthalmology, Wrocław Medical University. Sixty two age matched volunteers (19 males and 43 females) with no ocular and systemic pathologies were recruited from the university staff and their family members to form a control group (CG). The group of patients was further divided into four subgroups including those diagnosed:

(i) with the primary open angle glaucoma (POAG); diagnosis of POAG was based on glaucomatous changes in the optic nerve head with corresponding visual field defects and high or normal IOP in the presence of an open angle

(ii) with the primary angle closure glaucoma (PACG); diagnosis of PACG was based on glaucomatous changes in the optic nerve head with corresponding visual field defects in the presence of an anatomically narrow angle

(iii) as glaucoma suspects based on ocular hyper tension (OHT); diagnosis of OHT was applied to cases with IOP ≥ 21 mmHg, no glaucomatous changes in the optic nerve head, normal visual fields, and an open angle

(iv) as glaucoma suspects with glaucomatous optic disc appearance (GODA); diagnosis of GODA was determined by clinical assessment (narrowing of the neuroretinal rim with optic cup concentric enlargement, localized notching, or both), but normal visual fields and an open angle.

Subjects were fully informed of the purpose of the study and all procedures and their requirements. Informed subject consent was obtained before any measurements were taken. The project was approved by the Ethics Committee of the Wrocław Medical University (KB 481/2009) and adhered to the Tenets of the Declaration of Helsinki. The criteria for exclusion from the study were: any systemic disease, intraocular surgery less than six months before the study start date, refractive surgery, conjunctival or intraocular inflammation, corneal abnormalities such as edema or scars, and contact lens wear.

None of the subjects was taking any systemic medications. In the POAG and PCAG groups, patients were taking beta-blocker drops (27% and 30%, respectively), prostaglandins (36% and 32%), carbonic anhydrase inhibitor eye drops (28% and 19%) and alpha agonists (9% and 19%). Seventeen percent and 31% of patients were taking medications in the OHT and GODA group, respectively. Patients were taking beta-blocker drops (12% and 11%, respectively), prostaglandins (7% and 20%), carbonic anhydrase inhibitor eye drops (8% and 7%) and alpha agonists (1% and 3%).

A comprehensive clinical protocol was used in the following order:

[1] Review of general and medical ophthalmological history;

[2] Best corrected distance and near visual acuity;

[3] Central corneal thickness (CCT) measurement (PalmScan AP2000 A-Scan Biometer, MicroMedical Devices Inc., Calabasas, CA, USA) after instillation of one drop of 0.5% Alcaine (Proparacaine Hydrochloride, Alcon-Couvreur).

[4] Dynamic contour tonometry with anesthesia (DCT); continuous IOP pulse wave recordings were taken at a sampling rate of 100 Hz; the average (mean ± standard deviation) recording time of the IOP pulse wave for all subjects was 20.0 ± 4.6 seconds. Measurements were repeated until three IOP recordings with a quality score Q of three or higher were obtained [9,14,18-20]. Output data from the DCT included estimates of IOP, OPA, and HR.

[5] Goldman applanation tonometry; one drop of 2% Thilorbin (Fluorescein Sodium, Oxybuprocaine Hydrochloride, Alcon Pharma GmbH) was then entered into the conjunctival sac 30 seconds before the GAT measurement; three measurements were taken for the mean IOP value. GAT measurement was masked with respect to the measurement of DCT. Tonometry measurements were not randomized but sufficient time between measurements was allocated to avoid possible bias.

Additionally, after half an hour break, the glaucoma clinic patients underwent

[6] optic nerve head examination with Heidelberg scanning laser ophthalmoscope examination (HRT 3, Heidelberg Engineering, Heidelberg, Germany) after topical cycloplegia with sol. 1% Tropicamide,

[7] posterior segment examination with optical coherence tomography (sOCT, Copernicus, Optopol, Zawiercie, Poland)

[8] automated visual field examination using the Octopus 101 perimeter (Interzeag/Haag-Streit, Koeniz-Bern, Switzerland) were performed with dynamic test strategy (threshold algorithm, program G1).

**Figure 1:**
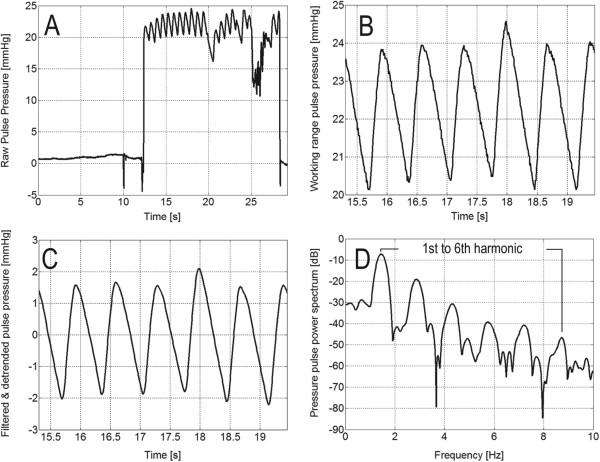
An example of the raw pulse pressure wave from Pascal DCT (A), the extracted working range wave (B), the detrended and filter wave (C) and the spectral content containing the first 6 harmonics (D).

DCT time series of the intraocular pressure pulse wave were saved and analyzed with custom written software, written in MATLAB (MathWorks, Natick, MA, USA), that included signal preprocessing, filtering and spectral analysis. The preprocessing step consisted of extracting the working range of the pulse pressure wave from raw Pascal data file (see Figure 1A and 1B) and removal of the signal trend (so-called detrending procedure). Signal filtering was achieved with the Savitzky-Golay filter [21] of order three with a smoothing window of length equal to 15 samples (see Figure 1C). Filtering raw DCT signal was necessary to increase the efficacy of subsequent spectral analysis. Spectral content of the IOP wave was obtained with a consistent power spectral estimator based on the windowed periodogram (fast Fourier transform, FFT). The Hamming window applied in the time domain was used. The FFT length was set to 4096 samples, regardless of the length of the measured signal to allow direct comparison between different spectral signal representations. Spectral content up to the 6th harmonic of the pressure pulse wave was considered (see Figure 1D).

Previous attempts of analyzing the spectral content of the intraocular pulse signals focused on estimating the amplitudes of each signal harmonic [16,17]. Such an approach is prone to errors due to inherent nonstationarity of signals caused mainly by the heart rate variability and respiratory sinus arrhythmia [22]. Nonstationarity means that the signal frequency spectrum varies in time [23]. To minimize this unwanted effect, two measures of spectral content were considered:

(i) the ratio of spectral harmonic amplitude of the IOP pulse wave and that of the first (principal) harmonic, *A*_*n*_*/A*_1_, *n* = 2, 3, …, 6.

(ii) the ratio of spectral harmonic energy of the IOP pulse wave and that of the first (principal) harmonic, *S*_*n*_*/S*_1_, *n* = 2, 3, …, 6, where the harmonic energy was calculated, using numerical integration, as the area of +/-0.2 Hz around the harmonic amplitude.

It should be noted that the proposed spectral analysis approach does not consider the effect of IOP level (signals were detrended) and the effect of OPA amplitude (ratios of amplitudes and energies were used).

Statistical analysis included standard descriptive statistic. All data were tested for normality using the Jarque-Bera test. Normality was rejected in the majority of cased (*p* < 0.05) as the data distributions were often skewed. Hence, multicomparison for independent groups has been performed using Kruskal-Wallis test (Statistica, ver. 10, StatSoft, Inc., USA).

## Results

The main group demographics together with the group mean GAT IOP, DCT IOP, OPA, HR and CCT are shown in Table 1. There were statistically significant differences (Kruskal-Willis test *p* < 0.05) between median ages within subject groups but the ranges were similar.

**Table 1:** Number of subjects, mean age, GAT IOP, DCT IOP, OPA, HR and CCT for the five considered groups

**Variables**	**CG**	**Suspects group**		**POAG**	**PACG**
		**GODA**	**OHT**		
Number of subjects (M/F)	62 (19/43)	43 (11/32)	31 (16/15)	197 (68/129)	25 (9/16)
Mean age (years ± SD) (range)	68 ± 11 (41 ÷ 87)	62 ± 14 (41 ÷ 86)	61 ± 11 (41 ÷ 81)	66 ± 11 (40 ÷ 86)	65 ± 9 (41 ÷ 75)
Mean GAT IOP (mmHg ± SD) (range)	14 ± 3 (8 ÷ 22)	15 ± 3 (10 ÷ 20)	21 ± 1 (19 ÷ 26)	16 ± 4 (9 ÷ 31)	16 ± 3 (10 ÷21)
Mean DCT IOP (mmHg ± SD) (range)	16.9 ± 2.6 (10.3 ÷ 24.3)	17.4 ± 2.3 (9.9 ÷ 21.0)	22.7 ± 2.8 (18.4 ÷ 31.8)	18.5 ± 4.2 (7.9 ÷ 39.7)	17.5 ± 3.5 (12.4 ÷ 46.9)
Mean OPA (mmHg ± SD) (range)	2.9 ± 1.3 (0.6 ÷ 6.6)	2.7 ± 1.1 (0.9 ÷ 5.3)	4.1 ± 1.6 (0.9 ÷ 8.5)	3.1 ± 1.4 (0.5 ÷ 9.2)	3.8 ± 1.3 (1.3 ÷ 6.8)
Mean HR (bpm ± SD) (range)	70 ± 8 (53 ÷ 94)	73 ± 11 (52 ÷ 109)	70 ± 11 (54 ÷ 115)	70 ± 8 (43 ÷ 172)	72 ± 14 (52 ÷ 97)
Mean CCT (μm ± SD) (range)	561 ± 43 (478 ÷ 670)	545 ± 33 (450 ÷ 595)	574 ± 31 (538 ÷ 665)	550 ± 40 (424 ÷ 648)	559 ± 28 (509 ÷ 621)

The results of a multiple comparison of medians using the Kruskal-Wallis test for GAT IOP, DCT IOP, OPA, HR and CCT are shown in Table 2. For GAT IOP significant differences (*p* < 0.05) were found between three pairs of considered groups, that is, between CG and OHT, GODA and OHT, and between PACG and OHT. For DCT IOP, significant differences were recorded for five out of 10 multiple comparisons. Significant differences (*p* < 0.05) were found between OHT and all other groups, and between CG and POAG. OPA showed statistically significant differences in seven cases. No significant differences were found between CG and GODA, CG and POAG, and between OHT and PACG. HR and CCT showed statistically significant differences in only two cases each.

**Table 2:** **The results of the Kruskal-Wallis test (*p*-values) for multi-comparison of medians of the five considered groups**

	**GAT IOP**				**DCT IOP**				**OPA**			
	**GODA**	**OHT**	**POAG**	**PACG**	**GODA**	**OHT**	**POAG**	**PACG**	**GODA**	**OHT**	**POAG**	**PACG**
CG	NS	**<0.001**	NS	NS	NS	**<0.001**	**<0.001**	NS	NS	**<0.001**	NS	**<0.001**
GODA		**<0.001**	NS	NS		**<0.001**	NS	NS		**<0.001**	**<0.050**	**<0.001**
OHT			**<0.001**	NS			**<0.001**	**<0.001**			**<0.001**	NS
POAG				NS				NS				**<0.001**
	**HR**				**CCT**							
	**GODA**	**OHT**	**POAG**	**PACG**	**GODA**	**OHT**	**POAG**	**PACG**				
CG	NS	NS	**<0.010**	NS	NS	NS	NS	NS				
GODA		NS	**<0.001**	NS		**<0.010**	NS	NS				
OHT			NS	NS			**<0.010**	NS				
POAG				NS				NS				

Statistically significant results (*p* < 0.05) are shown in bold font. NS denotes a statistically insignificant result.

For HR, statistically significant differences (*p* < 0.05) were found between CG and POAG and between GODA and POAG. Note that the fundamental frequency of the Pascal DCT signal, estimated using a spectral analysis method that takes into account non-stationarity of signals, corresponds very closely to the HR value given by Pascal instrument.

For CCT significant differences were found between GODA and OHT and between OHT and POAG.

**Figure 2:**
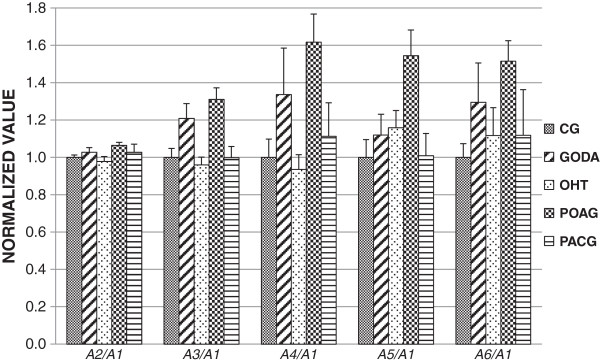
The ratios of spectral harmonic amplitudes of the IOP pulse wave and that of the first (principal) harmonic, normalized with respect to the control group average.

**Figure 3:**
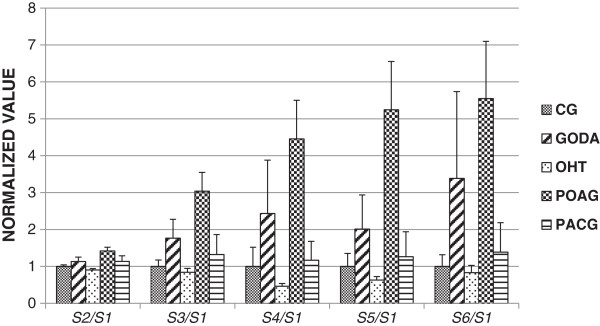
The ratios of spectral harmonic energies of the IOP pulse wave and that of the first (principal) harmonic, normalized with respect to the control group average.

Figure 2 shows the ratios of spectral harmonic amplitudes (*A*_2_, *A*_3_, *A*_4_, *A*_5_, and *A*_6_) of the IOP pulse wave and that of the first (principal) harmonic (*A*_1_), normalized with respect to the control group average. Correspondingly, Figure 3 shows the ratios of spectral harmonic energies (*S*_2_, *S*_3_, *S*_4_, *S*_5_, and *S*_6_) and that of the first (principal) harmonic (*S*_1_), normalized with respect to the control group average. Error bars in both figures indicate one standard error.

Separation trends between the groups were observed for both ratios of spectral harmonic amplitudes and energies, where this effect was more pronounced for the latter. Note that the considered spectral ratios do not indicate differences in the IOP and OPA levels but differences in the IOP pulse wave shape. The higher the harmonic frequency the larger the separation trend was obtained. This was, however, at the cost of larger noise, resulting in non-significant differences in the statistical analyses.

For the amplitude and energy based ratios, there was no statistically significant differences between any pair of considered groups using *A*_2_/*A*_1_, *S*_2_/*S*_1_, *S*_5_/*S*_1_ and *A*_6_/*A*_1_. Statistically significant differences (*p* < 0.05) were found between CG and POAG for *A*_4_/*A*_1_, *S*_4_/*S*_1_, *A*_5_/*A*_1_ and *A*_6_/*A*_1_. For the *A*_3_/*A*_1_ ratio, significant differences (*p* < 0.05) were obtained for CG and GODA and CG and POAG, while for the *S*_3_/*S*_1_ ratio, significant differences were obtained for CG and POAG, OHT and POAG, and between POAG and PACG.

## Discussion

It was of interest to ascertain whether spectral analysis of DCT signal could be clinically utilized to evaluate the shape of the intraocular pulse pressure wave in healthy eyes, glaucoma suspects (GODA and OHT), and glaucoma types (POAG and PACG).

Of particular interest was to determine whether differences in intraocular pressure pulse wave shape between the groups exist in glaucomatous eyes with sub-normal levels of IOP.

Evans and colleagues [16] and Božić and colleagues [17] showed statistically significant differences in the higher harmonic amplitudes of the IOP pulse wave spectra between healthy and glaucomatous eyes. Their subject group size was 10 and 20, respectively. We have modified their basic signal processing methodologies and considered amplitude and energy ratios because estimating harmonic amplitudes was found not to be robust when larger groups of subjects were considered.

Note again that spectral analysis of the IOP pulse wave describes the shape of the signal but not the level of IOP pressure (in mmHg), which is contained in zero frequency (constant signal value). This information is not considered in the spectral analysis because the signals were detrended. Additionally, the normalization of the higher harmonics to the first harmonic of the DCT signal makes the analysis results independent of the OPA value. Such a normalization is particularly important in studies that involve groups of subjects with varying IOP and OPA levels, such as considered by Božić and colleagues [17].

The calculated amplitude and energy ratios showed distinctive increasing trends with higher harmonics. However, substantial variability in the higher harmonics was observed. Of interest could be the results of the third harmonic ratios (both amplitude and energy). In the study of Evans and colleagues [16] the third harmonic also led to the smallest *p*-value, while Božić and colleagues [17], although considered spectral content up to the fifth harmonic, showed the results up to the third harmonic.

Worth noting is that spectral analysis of the pulse pressure signals was the only method among other measured parameters (GAT IOP, DCT IOP, OPA, HR, and CCT) that showed significantly different results between CG and GODA groups. On the other hand, the basic spectral analysis of Božić and colleagues [17] showed statistically significant difference between healthy eyes and those of POAG but not between CG and the normal tension glaucoma group.

## Conclusions

Spectral analysis techniques that take into account the non-stationary character of the DCT signals and are independent of IOP and OPA levels could prove to be useful for describing the characteristics of the intraocular pulse pressure wave distinctive to certain types of glaucomatous eyes or glaucoma suspects. In particular, such an approach showed the ability to distinguish healthy eyes from those of suspects with glaucomatous optic disc appearance. Until now, such discernment could have been performed examining the optic nerve head and retinal nerve fiber layer using more expensive devices such as the OCT, HRT, and scanning laser polarimetry (GDx).

Finally, it is worth noting that the proposed spectral analysis of intraocular pulse pressure wave has been designed in such a way to phase out any differences in the levels of IOP and OPA so that all five considered groups of subjects (two glaucomatous, two suspects, and the control) could have been compared.

## Competing interests

The authors declare that they have no competing interests.

## Authors’ contributions

MA-W is responsible for the design of the study, the study protocol, recruitment, co-ordination of resources, and drafting the manuscript. PK-B is responsible for the execution of the clinical protocol and diagnosis and treatment of patients. MK is responsible for the design of the study protocol. DRI is responsible for the design of the study, data analyses and drafting the manuscript. All authors read and approved the final manuscript.

## Pre-publication history

The pre-publication history for this paper can be accessed here:

http://www.biomedcentral.com/1471-2415/14/30/prepub
